# Structural stigma within inpatient care for people who inject drugs: implications for harm reduction

**DOI:** 10.1186/s12954-024-00971-6

**Published:** 2024-02-27

**Authors:** Maham Rehman, Leigh Chapman, Lisa Liu, Sara Calvert, Javeed Sukhera

**Affiliations:** 1 Department of Psychiatry, Schulich School of Medicine and Dentistry, Western University, London, Canada; 2 Inner City Health Associates, Toronto, Canada; 3 Present Address: Institute of Living Terry Building, Institute of LivingHartford Hospital, 200 Retreat Avenue, Hartford, CT 06106 USA; 4 Present Address: Northern Ontario School of Medicine, Sudbury, ON Canada

**Keywords:** Structural stigma, Inpatient care, Harm reduction, Substance abuse

## Abstract

**Background:**

Individuals suffering with addiction have historically experienced disproportionally high levels of stigma. The process of inpatient care for those with substance abuse disorder (SUD) is multifaceted, shaped by the interplay of human interactions within the healthcare team and overarching structural factors like policy. While existing literature predominantly addresses personal and interpersonal stigma, the influence of structural stigma on care delivery practices remains understudied. Our research aims to investigate the impact of structural stigma on care processes for individuals with SUD admitted to acute medicine units.

**Methods:**

We conducted a secondary analysis of observation notes and interview transcripts utilizing an analytic framework related to structural stigma adapted from previous research. Data was collected from June 2019 to January 2020 in 2 hospitals. 81 participants consented to observation and 25 to interviews. Interviews were conducted with patients (*n* = 8), healthcare staff (*n* = 16), and caregivers (*n* = 1).

**Results:**

Each aspect of care for people with SUD is adversely influenced by structural forms of stigma. There was evidence of a gap in accessing care and time pressures which deteriorated care processes. Structural stigma also manifested in the physical spaces designed for care and the lack of adequate resources available for mental health and addictions care. We found that structural stigma perpetuated other forms of implicit and explicit stigma.

**Conclusions:**

Structural stigma and other forms of stigma are interconnected. Improving care for people with SUD in hospital settings may require addressing structural forms of stigma such as how physical spaces are designed and how mental healthcare is integrated with physical healthcare within inpatient settings.

## Introduction

Individuals with substance use disorder (SUD) have historically experienced disproportionally high levels of stigma in healthcare spaces [1]. This impairs function, quality of life, and contributes to poor care outcomes [2]. Despite improvements over time [3], individuals with SUD, particularly people who inject drugs, continue to experience significant stigma in the healthcare system. They are continuously stereotyped, labelled, and subjected to discrimination [1]. People who inject drugs also experience significant social marginalization and mistrust in the healthcare system due to negative interactions with healthcare professionals and organizations. This produces inequitable healthcare outcomes for this patient population [3, 4].

Stigma is a concept that refers to the social and structural marginalization of individuals or groups with less than desired attributes. Stigma can be understood in terms of the different ways it manifests at the individual, social and structural levels. Social or interpersonal stigma is a phenomenon in which stereotypes relating to specific social groups are endorsed. Within it, health-related stigma describes a process in which social groups are devalued, rejected, and excluded based on a socially discredited label [5]. Social stigma is further perpetuated by structural stigma which refers to the ways in which rules, policies, and procedures of organizations or society at large restricts the rights and opportunities for specific groups [6].

Understanding various facets of stigma becomes especially intricate when considering individuals who inject drugs and their interactions within healthcare settings. For example, people who inject drugs are often perceived by health professionals in pejorative ways as ‘demanding’, ‘drug seeking’, and/or ‘non-compliant’ [7, 8]. These attitudes are often coupled with a lack of knowledge and skills among healthcare professionals to adequately care for this patient population. Ignorance and stereotypes can breed frustration and tension among healthcare professionals when they care for patients who inject drugs. Professionals often perceive treating this population as more 'challenging' and 'stressful'. They also express that the use of healthcare resources to treat 'self-inflicted' and 'repetitive problems' such as addiction is highly wasteful [10]. These stigmatizing attitudes significantly diminish the self-worth, help-seeking behavior, and treatment adherence of people who inject drugs, often leading to internalized self-stigma [1, 11].

Despite the importance of addressing stigma towards SUD, existing research on stigma has largely focused on investigating the concept from a cross-sectional approach, seeking to understand the attitudes and behaviors of patients and healthcare providers. Little attention has been given to understanding how structural forms of stigma may influence the enactment of care processes. Healthcare processes for people who inject drugs are complex and influenced not only by social interactions, but also by structural components at organizational and societal levels. Structural factors include but are not limited to hospital systems, policies, technology, time, and space [12, 13]. When considering the role of structural stigma for people who inject drugs, procedures, policies, and practices within health organizations and society at large can contribute to inequitable care [14]. Structural stigma can also be reflected in and include poor distribution of resources, withholding of services, undertreatment, and a systemic delivery of low-quality care [15].

Complex health organizations often provide care for people who inject drugs yet struggle to consider structural stigma and its influence on care processes. Further research on how structural stigma influences the care of people who inject drugs may provide a deeper understanding on the ways structural stigma is enacted in care processes. This can mobilize knowledge to identify and resolve stigmatizing aspects of care. In this study, we sought to explore how structural stigma influences the care of people who inject drugs admitted to acute hospital settings.

## Methods

The authors performed a secondary analysis of qualitative data from a previous study. This study sought to understand the process of care for patients who inject drugs, specifically focusing on attitudinal and behavioural components of care from a sociomaterial perspective [16].

Data consisted of detailed field observation notes, artifacts, hospital policies, and field interviews with a total of 81 participants. Data was collected from general medical units across two acute-care teaching hospitals in a mid-sized Canadian city from June 2019 until January 2020. Participants included patients who inject drugs *(n* = 10), their caregivers *(n* = 1), physicians *(n* = 7), medical learners *(n* = 46), nurses—including registered practical nurses, registered nurses, nurse practitioners *(n* = 12), and other health disciplines including a social worker, patient care facilitators, a pharmacist, physiotherapist and occupational therapist *(n* = 6). Healthcare staff were recruited through email invitations outlining the purpose, design, and methods of the study. Email invitation lists included staff working in the acute medicine ward such as the clinical teaching unit. Patients were recruited through identification. Participating nurses informed the research team when a patient who inject drugs was admitted into acute care allowing them to approach the patient for consent. Verbal and written informed consent were obtained in person from both healthcare providers and patients/caregivers at the time of observations or interviews.

81 participants consented for observation. Members of the research team (SC and LL) collected detailed and reflective notes on observation of clinical care encounters. This included notes on the healthcare team’s interaction with the patient, caregivers, and each other. Semi-structured interviews were conducted with 25 participants including patients (*n* = 8), caregivers (*n* = 1), and healthcare staff (*n* = 16). Interviews lasted 30–45 min. The questions in the interview guide were developed by the research team. The semi-structured interview guide was revised iteratively. Interviews aimed to undertsand (1) Patients experiences in acute care settings and (2) Healthcare professionals’ perceptions of patients with substance use disorder (see Appendix 1).

During the course of the initial study, the research team found that structural stigma appeared to have a unique influence on care processes. Each of the identified patient cases were influenced by structural factors, which often arose in discussions on discharge planning, follow-up care, and pain and withdrawal management. We therefore felt it would be important to conduct a secondary content analysis of observational data with a specific focus on *how* structural factors may have influenced care processes in dynamic and overlapping ways. Therefore, we conducted a secondary analysis with a specific focus on structural forms of stigma, to explore how the quality-of-care people who inject drugs receive may be influenced by structural stigma embedded within policy and practice.

We conducted a secondary latent content analysis of observation notes and interview transcripts from a pre-existing data set. Data was stored in a password protected spreadsheet and organized based on type of data (e.g., observation or interview transcript). We re-read interview transcripts and observation notes line by line and identified instances in which structural factors were present [17]. In conducting qualitative latent content analysis, we systematically identified words, phrases, and concepts within the dataset and organized them into emerging themes pertaining to structural stigma to establish the initial coding framework [18]. We subsequently reviewed and refined these themes in alignment with the Mental Health Commission's framework on structural stigma, which delineates potential indicators, measures, and audit items [19]. The resulting framework represents our conclusive coding structure (Table 1).

**Table 1:** Indicators guiding analysis of transcripts

Indicator	Definition
Financial	Is there evidence of discrepancy in financial allocation for care? If structural stigma is reflected in resource allocation, how does it influence the practices of those delivering and receiving care?
Infrastructure	Is stigma reflected in the physical spaces where care is delivered and how such spaces are designed?
Decision making	Does structural stigma manifest in the ways that patients are involved in care decisions? Is there evidence of structural stigma influencing coercive practices?
Triage	Is structural stigma reflected in triage policies and practices?
Access and follow up	Is structural stigma reflected in access to care and access to follow-up care
Screening and assessment	Is structural stigma reflected in screening and assessment of PWID patients compared to non-PWID patients?
Education and skills	Is structural stigma manifested in the skills and training of health professionals to care for PWID

One researcher (M.R.) was responsible for the primary analysis, and a second researcher (L.C.) independently conducted a similar process and cross-checked the primary reanalysis findings. They resolved differences by discussion and consensus. Coding samples were reviewed with JS to check for accuracy and further refined. Coding continued to be refined throughout analysis through discussion with the entire team until theoretical sufficiency was achieved. This is when the study team agreed the analysis had adequate conceptual depth, was conceptually plausible, and was externally relevant to the broader academic community [20].

Our interprofessional team included one practicing physician, a child and adolescent psychiatrist (JS) and director of an overdose prevention site (LC)—each with extensive experience in qualitative research. JS is an expert on stigma and bias and LC is an expert on substance use disorder/harm reduction. The team also included an undergraduate Research Associate (MR), a registered nurse (SC), and a medical student (LL). MR conducted data analysis and performed coding with regular consultation and discussion with JS and LC. All provided input into study design and methods with guiding resources provided by the senior author. Our team included individuals who were acquainted with a primarily medical paradigm while also having knowledge of community-based harm-reduction services. The entire team was involved in synthesizing data and manuscript preparation.

## Results

Throughout our analysis we found that each step within the process of care for people who inject drugs was influenced by structural forms of stigma that dynamically interact with interpersonal forms of stigma. This was reflected through interactions between healthcare professionals and people who inject drugs. We organized our findings in four overlapping categories: access to care, financial and infrastructure resources, clinical decision making, and follow-up care planning. Gaps in access to care were influenced by infrastructure and financial limitations at the health system levels. Structural forms of stigma relating to poor access and restricted funding influenced stigmatizing decision-making practices such as inappropriate pain and withdrawal management for patients who inject drugs. Structural forms of stigma also impaired delivery of care interventions and compromised follow-up care and discharge planning. Overall, these gaps produced inequities in the process of care for people who inject drugs and contributed to social forms of stigma including blame and mistrust.

### Access

As the first step in the process of care, we defined access to care as the availability of healthcare services for people who inject drugs, specifically in relation to wait times and admission into acute-care settings. In acute-care settings, we noted that health professionals face expectations of efficiency and urgency, placing considerable pressure on them to deliver care efficiently to individuals who inject drugs. In one case, a healthcare professional expressed the need to “go fast [through the patient list] [8_transcript_ObsNotes].” A sense of needing to rush was also expressed by other health professionals stating, “I have other patients [to see] [1_transcropt_HCP001],” and that “time is a limited resource [8_transcript_HCP].” When health professionals in the field were asked about the underlying factors that contributed to their perception of the need to be efficient, some attributed this practice to mandates on discharge and admission rates. However, professionals also expressed that the nature of care in acute-care settings allows for “shorter time to deal with patients as [compared to longitudinal disciplines] such as family medicine [2_transcript_HCP003]”. While this sentiment generally influences the care provided to all patients regardless of substance use status, our observations revealed a concerning discrepancy. Individuals who inject drugs received notably shorter periods of allocated care which was justified by the healthcare team as a response to patient census demands. This is evident from a patient perspective. Our analysis found that professionals' urgency to be efficient produced gaps in the quality of care that people who inject drugs received. People who inject drugs frequently reported “being treated like a second-rate citizen [2_transcript_PT02]”, “having to beg, borrow, and fight…[for] the littlest thing including the healthcare teams time [10_transcript_PT03],” and having to prove “how sick you really are [10_transcript_PT03].” The time pressures experienced by healthcare professionals in acute-care settings compromised the treatment process for people who inject drugs, limiting their access to care. For example, in one case a patient’s echocardiogram appointment was delayed due to poor triaging by the healthcare team, hindering the patient’s access to essential antibiotics. In another patient case, the nurse's workload constraints rendered them occupied, thereby limiting the patient's ability to go outside. Consequently, the patient chose to leaveingainst medical advice. [12_transcript_ObsNotes].

### Financial and physical infrastructure

Structural stigma also manifested through how funding and resources influenced care. Financial factors included budget allocation, availability of resources, and staffing. Healthcare professionals often characterized caring for people who inject drugs as “wasting taxpayers’ money [9_transcript_F001].” When prompted to delve into these attitudes, healthcare professionals conveyed that providing care for this patient population necessitates expenditure of “hundreds of dollars [9_transcript_F001],” elaborating that this due to the recurrent cycle of admission and readmission because of the "harmful behaviors these patients engage in [1_transcript_HCP001].

We also found that short staffing was common on the acute-care floor. In one instance, a patient's procedure faced delays due to the nurses on duty being overwhelmed with a higher than usual caseload. The healthcare professional involved lamented that 'the model has changed … there was a reduction in budget [and as a result] every nurse on the floor was responsible for more patients than previously [1_transcript_HCP001].' Likewise, we found several instances where healthcare teams were inundated with numerous patients on their consultation list, driving them to prioritize speed, thus intensifying the sense of urgency and time constraints.

In conjunction with financial factors, physical infrastructure was another example of structural stigma influencing processes of care. Physical infrastructure included the ability to move freely through and outside of hospital property, presence or absence of security personnel, and access to staff. In this context, structural stigma and interpersonal forms of stigma dynamically interacted to produce poor outcomes for patients who inject drugs. For example, we found that health professionals' attitudinal perception of people who inject drugs translated into behaviors that were influenced by physical infrastructure. For instance, a general mistrust of patients who inject drugs, contributed to increased surveillance of these patients on acute-care floors. Patients described “being watched, [and] having security posted outside [their] door [2_transcript_PT02]. Patient stated that they were “not allowed to leave the hospital property [10_transcript_Pt03],” and used terms such as ‘escape’ and ‘prison’ when describing their care experience. The descriptors used by people who inject drugs suggest feelings of confinement and disempowerment in care spaces, indicating that the way physical spaces are designed and implemented affects healthcare experiences. This finding illustrates the presence of spatial stigma when caring for people who inject drugs, such that the social identity of someone with SUD affects the physical characteristics of their care space [21, 22]. In addition to feeling monitored, people who inject drugs were often absent from care spaces due to their desire for a break. This was often perceived as ‘oppositional’ or ‘behavioral’ by staff, contributing to neglect, avoidance, and poor care outcomes.

### Care plan development and delivery

In addition to lack of financial and physical resources, structural stigma also manifested in a discrepancy in the skill sets that health professionals utilized to provide adequate care for patients who inject drugs.

Significant deficiencies and shortcomings were noted among the healthcare team in relation to skill level, knowledge of addiction and substance use, and experience working with patients who inject drugs. Several participants outlined the “challenge [of] managing the skill mix” [1_transcript_HCP001] especially for members of the team who were “starting just out of school” [1_transcript_HCP001]. Observation notes indicated that many staff assigned to care for people who inject drugs were junior healthcare professionals and their lack of prior experience and minimal understanding of addiction was a strong contributor to how care decisions were made with people who inject drugs. In one case, the resident physician was hesitant to prescribe oral antibiotics to a patient citing worries that the patient would sell them once discharged. From our observation of this case, conversations between the team and the patient did not create reasonable suspicion the patient would sell these medications and the patient was previously cleared by the addictions care consultant [2_observation notes]. In another case, the junior resident was unfamiliar with pain and withdrawal management practices for people who inject drugs, resulting in inadequate pain medication provision. Moreover, one junior healthcare professional expressed that limited guidance and a lack of “clear clinical pathway to help manage this population [9_observation notes],” citing that this made the treatment process for people who inject drugs challenging.

A lack of adequate knowledge and skills also influenced care processes in structural ways. We often found that the physical health of patients who inject drugs was addressed while their mental health or social needs were often ignored. For instance, one healthcare professional clearly expressed that “we treat the acute medical issues and then patients are responsible to find their own housing… our social worker would provide the means for providing the information of where the shelters are [6_transcript_HCP005].” Similarly, other healthcare professional participants expressed that “[we] don't necessarily come up with as thorough a care plan every time [17_transcript_HCP014]” and that care for people who inject drugs is complex “because of their social situation and all the kind of social factors around them [21_transcript_HCP015].” From a people who inject drugs perspective, patients reported that their needs were not adequately addressed by the care team. For example, one patient cited the “need for… [their]ADHD medication… I was told very bluntly yesterday that they don’t really care about those things. They just want to focus on my infection [15_transcript_PT008].” Moreover, patients expressed the desire for healthcare professional to “broaden their horizons with other medications [to address] my mental health…but they want to specifically just tend to my infection [15_transcript_PT008].” These comments suggested that the structural stigma was deeply embedded within a medically focused model of care which consistently neglected mental health and the social needs of patients.

### Discharge and follow-up

The final step in the process of care involved appropriate follow up and discharge planning. Our analysis found the presence of limited discharge planning. Initially we observed this was fueled by time pressures and the urgency to increase the available beds. However, analysis of participant transcripts revealed that people who inject drugs were often discharged prematurely. For instance, in one case the healthcare provider expressed discharging the patient who injected drugs “as soon as possible… if any tests come back abnormal, we will bring [them] back [10_transcript].” Similarly, observation notes from another case illustrate that the healthcare team wanted to discharge the patient, however because of concern for an n infectious disease the patient needed a consultation. The healthcare professional on the team claimed, “they’ll discharge [them] regardless [11_transcript].” A very similar example was found in another case were an attending physician wanted to discharge a patient who injected drugs before a procedure. We commonly found that people who inject drugs would be discharged before the completion of important aspects of their care such as diagnostic tests. Overall, lack of proper discharge planning was another example of a structural limitation in the process of care.

We also found poor follow up coordination for people who inject drugs. Health professionals often reported that people who inject drugs are “more difficult to follow up with, because they don’t have a permanent place, a lot of them don’t have phones… they don’t have income [6_transcript_HCP005].” Similarly, another healthcare professional expressed that ‘[People who inject drugs] don’t have a family doctor or set pharmacy… [they] don’t even have a set address [3_transcript_HCP004].” Another professional outlined that “we know from experience that we can't complete any antibiotic therapy for them, as an outpatient, because whatever port they have [potential to use and inject drugs using it] and because of their social situation, and all the kind of social factors around them [21_transcript_HCP15].” Participants often assumed that people who inject drugs would be “lost to follow up [21_transcript_HCP15]” and “the team has no idea what happens to [the patient], not even if they’re alive or deceased [9_transcript_F001],” leading to avoidance and neglect. As a result, discharge and follow-up needs for patients were often inadequately addressed. There was little to no consideration of detailed aspects of discharge planning such as patients housing status, medication coverage and transportation access to follow up care. These behaviors persisted, even with the knowledge that lack of adequate follow-up care worsened acute-care presentations in the future and increased the likelihood of readmission.

From a people who inject drugs perspective, patient interviews and observation notes indicated that people who inject drugs were often discharged prematurely and without a follow-up care plan. One patient highlighted that the healthcare team “discharged me so in order for me to get back in [to receive care] I had to go to emergency again [12_transcript_PT005].” The patient elaborates, citing that “they sent me home with antibiotics… and I couldn’t do it all on my own and then missed a bunch of doses, so I had to be readmitted into hospital [12_trasncript_PT005].” Similarly, another patient expressed that they were discharged and told “if it comes up positive, then we’ll call you and get you to come back [11_transcript_PT04].” Ironically, it has been cited in the literature that the care for people who inject drugs is complex due to the repetitive cycle of admission and discharge; however, this finding illustrates that healthcare professionals poor discharge planning may be a contributor to this phenomenon. Ultimately, gaps in discharge and follow up care planning for people who inject drugs were structural in nature.

## Discussion

Our analysis suggests that structural stigma is embedded throughout care processes for people who inject drugs, leading to significant gaps and adverse outcomes for this vulnerable patient population. Gaps in access to care coupled with financial limitations and mixed skill set among professionals compromises the implementation and development of an appropriate care plan for patients who inject drugs. We found that during admission to acute care unit patients who inject drugs are discharged prematurely, have limited access follow up care, and are not given adequate mental health support. These observed manifestations of structural stigma contribute to poor care outcomes, increase patient’s distrust of healthcare spaces, and perpetuate a cycle of admission and discharge..

### Different forms of stigma are dynamic and intersecting

Throughout our analysis, we found that negative attitudes toward people who inject drugs and various manifestations of structural stigma at the health system levels were dynamic and overlapping. For instance, from an infrastructure perspective we found that professionals' mistrust of people who inject drugs and their perception of these patients as “so called drug seeking,” translated into greater surveillance and restriction of movement. A similar narrative is evident in relation to decision making and care plan development. We noticed that varying skill levels among staff can hinder the comprehensive addressing of mental healthcare and the social aspects of addiction. The presence of blame-centered attitudes towards people who inject drugs underlies this observation, leading to tensions in the decision-making process. Moreover, premature discharge and poor follow up care planning are often attributed to the complex social situation of people who inject drugs (e.g., no access to a permanent address, family doctor, or pharmacy). Yet, undertones of bias and frustration with people who inject drugs perpetuated this stigmatizing process of care by contributing to a sense of futility. These findings illustrate that structural stigma is rooted in human factors, emphasizing that different forms of stigma are interconnected.

The interactions between different forms of stigma build upon our current understanding of stigma towards people with SUD in healthcare settings. Often discussions on stigma focus specifically on the level of the individual (patient, nurse, healthcare professional, etc.), linking individual and interpersonal stigma [23]. For example, there is an abundance of literature that explores barriers towards addressing stigma for people with SUD focusing on the role of healthcare professionals' attitudes and perceptions [23, 24]. The majority of the literature outlines healthcare professionals' negative attitudes as the primary source of stigma towards people with SUD, while describing how interpersonal stigma influences care delivery and treatment outcomes [25, 26]. However, there is rarely an analysis that explores how different levels of stigma are connected. Consequently, our study provides a unique example of how stigma research can be enhanced through observational approaches. We encourage researchers to conceptualize systems of stigma as interconnected, promoting interrogation of these connections to understand how stigma is reproduced offering guidance on approaches to addressing it.

### Implications for addressing structural forms of stigma in hospital settings

Our findings provided specific examples of structural stigma while offering implications for how to address them. For example, further investigation of physical spaces of care and how they are designed can make a significant difference. Our analysis reveals that people who inject drugs do not feel safe and comfortable in care spaces. Patients describe feeling like a ‘prisoner’ or being ‘trapped, indicating the presence of spatial stigma. Spatial stigma is rooted in structural factors, such as the social characteristics of an individual influence's physical infrastructure and behavior [21]. For people who inject drugs physical infrastructure and design heightens identification and experiences of stigma. Meaning, when a patient who inject drugs is constantly surveilled on an acute-care floor they become more aware that their social identity warrants distrust in healthcare spaces. Meaning constant surveillance of and distrust of people who inject drugs can impact care implementation and compromise the quality of the treatment relationship.

Spatial design informs the quality of our environment. Poorly designed spaces have poor technicality and functionality [27]. A care space, especially for stigmatized patient populations, must be designed to humanize, protect, and empower [27]. Thus, moving forward, care spaces needed to be designed in partnership with people who inject drugs. For example, Novotná and colleagues [28] evaluated a substance use treatment facility before and after implementation of client-centered design recommendations. The authors found that changes to physical design, based on recommendations by the clients of the facility had positive outcomes. Implementation of private rooms restored feelings of autonomy and empowerment to improve recovery. Greater investment in interior design and architecture, including an open-concept and friendliness of shared spaces, helped reduce stigma [28]. Thus, we suggest the need to consider physical infrastructure when optimizing care for stigmatized patients. Redesign of acute-care spaces should aim to optimize clinical services, eliminate feelings of surveillance, and empower people who inject drugs while also ensuring that service delivery and quality of care are not compromised [28, 29]. This requires interdisciplinary partnership between healthcare professionals, patients, and spatial designers, to identify specific needs and explore different infrastructure designs [29].

Another potential implication from our work involves the importance of integrating the provision of mental healthcare within acute hospital units. Several patient participants in our study expressed the desire for the healthcare professional team to address both the acute medical issue and their mental health concerns. If healthcare professionals do not consider the manifestations of addiction and how it influences diagnosis, differentials, and treatment, the delivery of care is compromised. For example, if a patient who injects drugs is admitted into an acute care for a soft tissue infection, treating with antibiotics without addressing the patient's withdrawal symptoms decreases the patient’s sense of safety in the care space. This can thereby limit patient compliance and increase the likelihood of patient absence in the care process. Fundamentally, this study highlights that failing to address the social and psychological facets of addiction is harmful and compromises the delivery of care. Given examples of how mental healthcare can be integrated to improve the delivery of both ambulatory and acute hospital care [30, 31] it is concerning that we found negligence of mental healthcare for people who inject drugs. Mental healthcare must integrate into basic processes when caring for all patients, but especially for people with SUD, given that addiction is a mental illness that impacts both mental and physical well-being [33]. This can occur in a variety of ways. For example, one study sought to evaluate a collaborative mental health evaluation framework in local acute care settings in Mexico, Nicaragua, and Chile [32]. The framework was found to be beneficial in determining the types of services needed by patients improving preliminary outcomes, specifically for patients with addiction. Another study implemented a job rotation program for nurses incorporating preceptorships in mental health [31]. Preliminary evaluation found that participation in the program increased nurses’ confidence, knowledge on mental healthcare, and better equipped them to identify the mental health needs of their patients. Examples of specific interventions include adding mental health professionals to care teams, screening for mental health concerns upon admission, and implementation of addiction services as well as safe-consumption spaces [30, 34].

### Addressing structural stigma through integration of harm reduction

Our findings suggest that structural forms of stigma interact with interpersonal stigma to compromise the quality-of-care people who inject drugs receive in hospital settings. Several of our findings align with the philosophy and practice of harm reduction, which refers to reducing harms associated with specific behaviors through compassion and non-judgmental approaches [35]. Harm reduction aims to reduce harm associated with a specific behavior without necessarily reducing the harmful behavior itself [36]. Harm reduction can be strategically integrated by hospital organizations to address structural stigma. This can be done through education and integration of safe-consumption spaces.

Our analysis found several instances where knowledge on addiction and skills in caring for people with SUD were lacking. For example, in several patient cases, healthcare professionals did not develop follow-up care plans for people who inject drugs because the patient’s social situation presented numerous challenges. Harm reduction can be utilized to address these shortcomings by integrating structural competency training for professionals working in acute care settings. Structural competency refers to the ability of health care professionals to recognize and respond to the social determinants of health that produce or maintain health disparities [37, 38]. Structural competency allows healthcare professionals to identify the role and influence of social factors in clinical experiences and have the skillset to address these vulnerabilities. For example, when discharging a patient who injects drugs without a permanent address, rather than expecting the patient to find housing themselves, a structurally competent healthcare professional would work with the patient and allied healthcare to find appropriate housing options. This professional would be aware of the benefits of safe and stable housing for the patients' general wellbeing. Moreover, a structurally competent healthcare provider would also seek to understand the patient's social situation, such as access to a pharmacy, transportation, and housing, and use this information to inform an accessible follow-up care plan.

Another example of integrating harm reduction into the process of care for people who inject drugs relates to integrating addictions care and safe-consumption sites into hospital organizations. Addiction medicine teams are often multidisciplinary. They seek to form partnerships with patients to understand their addiction needs and work to incorporate harm reduction into care processes [39]. These teams are highly effective in addressing withdrawal management, improving treatment compliance, and fostering positive treatment relationships [39, 40]. Addiction medicine teams have shown significant benefits in terms of mortality reduction and cost-effectiveness, leading to their widespread adoption in care centers [40]. They also have positive effects on healthcare professionals' attitudes towards addiction while also increasing their knowledge about addictions treatment [41]. Beyond addiction medicine-focused teams, our study unveiled tensions between healthcare providers and patients who inject drugs, particularly concerning ongoing drug use during hospitalization. Healthcare staff were frequently frustrated by patients' absence from the ward, while patients were frustrated that their addiction needs were not acknowledged or addressed by providers. Safe-consumption sites offer a reasonable option to address this gap. Safe consumption sites are supervised injection spaces that provide a hygienic environment for drug consumption under the observation of trained staff [42, 43]. They limit the spread of infection, overdose, and needle sharing. They are a common form of community-based harm reduction that has been strongly advocated for in acute-care settings [42]. People with SUD when admitted into hospital will continue to inject and consume drugs, thus healthcare delivery must focus on mitigating side-effects of drug use [43]. Safe consumption sites represent a highly effective intervention, but their successful implementation requires establishing a regulatory framework, ensuring suitable infrastructure, and securing adequate financial resources.

Ultimately, our research reveals that stigma manifests in various dynamic and interconnected forms. To combat structural stigma against patients with substance use disorder, prioritizing structural competency and integrating harm reduction principles within acute care settings is imperative. Achieving these objectives necessitates a deep understanding of the socialized healthcare system in Canada, wherein financial constraints may impede innovation.

### Limitations

Our analysis is limited by the onset of the COVID-19 pandemic which shortened the data collection period. There is a potential for selection bias in our study, as the majority of healthcare workers sampled were medical learners, specifically residents. The perceptions and attitudes expressed may not apply to all healthcare professionals. Our study was conducted in one healthcare organization in a mid-sized Canadian city. Moreover, our study is a reanalysis of primary data from a structural stigma perspective, we did not directly investigate systems of structural in the primary study. The study was also conducted in Canada which has a socialized healthcare system which limits financial barriers to accessing healthcare. Therefore, we caution the generalizability and transferability of the findings. However, this study highlights alarming trends on the role of stigma in healthcare delivery for people with SUD prompting greater investigation.

## Conclusion

Overall, this study illustrates that stigma is highly complex and rooted in both attitudes and structures. Stigma is often conceptualized in relation to attitudes of healthcare professionals, we build upon this ideology to illustrate the presence of policies and practices that perpetuate structural stigma and contribute to attitudinal versions of stigma. In an era where we are witnessing a rise in SUD it is vital that we interrogate systems of stigma and their interconnections to undertsand how the process of care can be compromised. This requires centering the voices of people with lived experience and partnering with experts in addictions care to translate our knowledge into tangible interventions.

## Data Availability

Transcripts annotated by theme available upon request. Coding available upon request. Please contact Maham Rehman (rehmanmaham@yahoo.ca).

## Appendix 1: Interview guide

### Healthcare professional

Q1. Describe what it's been like taking care of somebody who has substance use disorder, specifically injects drugs

Q2. What are the biggest barriers in providing care to this population?

- Follow up on the barriers.
  o Ask for specific details or examples

Q3. Do you feel equipped to address these barriers?

- Personal skills
- Resources
  o Staffing
  o Finance
- Time

Q4. Patient case specific questions

- Tell me about this patient
- How is your relationship with the patient

## Patient

Q1. Details about stay

- How long have you been admitted to hospital
- If you feel comfortable, what are you admitted for
- Summarize hospital stay

Q2. How is your hospital stay

- Are you satisfied with the care
- Describe your relationship with the care team
- Any challenges and barriers

Q3. Previous hospital admissions

- Have you been admitted before
- What was your experience like then, compare it too now

**COREQ Checklist—attached**
